# A School-Based Exercise Intervention Program Increases Muscle Strength in Prepubertal Boys

**DOI:** 10.1155/2010/307063

**Published:** 2010-06-22

**Authors:** Susanna Stenevi-Lundgren, Robin M. Daly, Magnus K. Karlsson

**Affiliations:** ^1^Clinical and Molecular Osteoporosis Research Unit, Department of Clinical Sciences, Lund University, 22100 Lund, Sweden; ^2^Department of Orthopedics, Malmö University Hospital, 205 02 Malmö, Sweden; ^3^Department of Medicine, Western Hospital, The University of Melbourne (RMH/WH), Footscray, Melbourne, Australia

## Abstract

This prospective controlled intervention study over 12 months evaluated the effect of exercise on muscular function, physical ability, and body composition in pre-pubertal boys. Sixty-eight boys aged 6–8 years, involved in a general school-based exercise program of 40 min per school day (200 min/week), were compared with 46 age-matched boys who participated in the general Swedish physical education curriculum of mean 60 min/week. Baseline and annual changes of body composition were measured by dual energy X-ray absorptiometry (DXA), stature, and body mass by standard equipments, isokinetic peak torque (PT) of the knee extensors, and flexors at 60 and 180 deg/sec by computerized dynamometer (Biodex) and vertical jump height (VJH) by a computerized electronic mat. The annual gain in stature and body mass was similar between the groups whereas the increase in total body and regional lean mass (*P* < .001) and fat mass (*P* < .001) was greater in the exercise group. The one-year gain in body mass-adjusted knee extensor and flexor PT at 180 deg/sec was significantly greater in the intervention group compared with the control group (*P* < .01, adjusted for age at baseline and *P* < .001, adjusted for age and muscle strength at baseline, resp.). There was no group difference in VJH. In conclusion, the increase in school-based physical education from 60 to 200 minutes per week enhances the development of lean body mass and muscle strength in pre-pubertal boys.

## 1. Introduction

Physical activity has been regarded as one of the most important life style factors that could improve a variety of health-related aspects, including musculoskeletal health. But studies have indicated that children and adults in the recent decades have become less physically active [1], and there is a growing concern that the more sedentary lifestyle might lead to increased obesity and increased risk factors for a variety of chronic diseases and fractures. In fact, some authors even infer that we should change our focus from improving bone mass to improving neuromuscular function through increased physical activity when trying to reduce the incidence of fractures, this through a reduction in the fall frequency [2]. 

Physical activity includes a variety of activities as it is defined as any bodily movement produced by skeletal muscles that result in energy expenditure [3]. Physical activity can thus be seen as a summary of different behaviour, including subcategories such as exercise, sport, leisure activities, dance, and transportation [4]. Exercise or training however is defined as a subset of physical activity that is planned, structured with repetitive bodily movement done as to improve or maintain one or more components of physical fitness [3]. Currently it is recommended that all growing children daily should participate in at least 60 cumulative minutes of moderate to vigorous physical activity that is developmentally appropriate, enjoyable, and includes a variety of activities [5], and at least 3 days per week should include activities of vigorous intensity [6]. In addition, recent recommendations add that specific bone and muscle strengthening activities each day ought to be included as to improve health [6].

In children and adolescents, it is well established that gains in strength and power are possible following prospective controlled short-term progressive resistance training programs two to three times per week. Reports show that muscle strength, muscle mass, and physical performance improved in both boys and girls [7–9]. There are also other health-related benefits that are associated with strength training, such as improved self-satisfaction, self-esteem, and body image [10]. Muscle mass, muscle strength and endurance are also significantly higher in young athletes than in sedentary controls [11–14] while the proportion of body fat usually is reported lower in athletes than in controls [14, 15]. 

To our knowledge there is not an extant literature on general exercise training of longer duration and its effect on neuromuscular development in young children. However, reports show positive effects on fat mass, physical fitness, and performance. A school-based program with expanded physical education lessons (4 lessons/week) during three years was effective in increasing children's physical performance and preventing excessive weight gain [16]. Similarly Ara et al. report that regular participation in at least 3 hours per week of sports activities as well as the compulsory physical education program for one year is associated with increased physical fitness, vertical jump height and lower body fat mass in Prepubertal boys [17]. After follow-up three years later, the physically active boys had increased their total lean mass to a greater extent and maintained their physical fitness during growth compared with controls [18]. Furthermore, during one year of regular sport-specific training young athlete boys increased their physical performance compared with untrained youth [19]. 

These studies provide important information about the musculoskeletal effects of resistance training programs in volunteers or of exercise training programs of longer duration but with obesity or physical fitness as main outcome. However, it still remains unclear whether a long-term, general, and moderately intense exercise program on population level could improve muscle mass and performance in children 6–8 years old. We have previously reported that this goal could be reached in Prepubertal girls [20] but there has been less evaluation of whether the same could be achieved in boys who are already more physically active during their spare time before the exercise intervention [21]. 

A population-based general exercise intervention program of moderate intensity was created by increasing the frequency of compulsory school physical education, and not the intensity, as to be able to include all children in the intervention, not only those who could stand a more high intense training. This study was designed to evaluate whether this intervention program could improve body composition, lower extremity muscle strength, and physical performance in Prepubertal boys. We hypothesized that the 12-month program would confer these benefits.

## 2. Materials and Methods

The Malmö Pediatric Osteoporosis Prevention (POP) Study, is a prospective controlled exercise intervention study designed to annually assess skeletal and muscle development in children from school start onwards [21, 22]. Baseline measurements in the intervention group were performed in August and September, just after school started and before the intervention was initiated. The follow-up evaluations were done in the same months one year later. The controls were evaluated in November and December with all follow-up measurements done during the same months but two years later. Annual changes (per 365 days) were then calculated for all measured parameters. The design was accepted as the literature suggests that bone mass, muscle mass, and muscle strength increase in a linear fashion during the Prepubertal period [21, 23–25], a design approved in previous publications [20–22, 26]. During the summer period, all children had a break for nine weeks when no additional exercise training was provided. 

The study design has previously been reported in detail when reporting changes in bone mass [21], but in summary, all boys in grades 1 and 2 in one school in Malmö, Sweden were chosen as intervention group. Of the 89 boys, 84 agreed to participate (94% inclusion). Two boys were excluded as they were on medications known to affect bone metabolism, and at follow-up one boy declined participation, leaving 81 boys with measurements both at baseline and at follow-up. After statistical descriptive analysis, 7 boys were excluded for having extreme values, defined as >3 standard deviations (SD) above or below the mean, and 6 boys due to technical measurement errors, leaving 68 boys with a mean ± SD age of 7.8 ± 0.5 years (range 6.7–8.6) at baseline to be included in this report. The controls were volunteers from three neighboring schools in areas with a socioeconomic background similar to that of the intervention group. Sixty-eight boys agreed to participate at baseline. At follow-up, 9 had moved out of the region or declined further participation, and 1 was excluded due to medication known to affect bone metabolism. After statistical descriptive analysis, 7 boys were excluded for having extreme values and 5 due to technical measurement errors, leaving 46 controls with a mean ± SD age of  7.9 ± 0.6 years (range 6.7–8.9) at baseline to be included in this report. All participants were healthy and all Caucasians, except one boy adopted from Colombia. 

In order to ascertain whether there was any selection bias at baseline, we have previously reported that there were no significant differences in the grade 1 examination regarding stature, body mass, and BMI of the boys, when a drop-out analysis compared the study participants and the nonparticipants [27]. Furthermore, there were no differences in baseline age, stature, body mass, BMI, total body or regional body composition, muscle strength, or vertical jump height between the boys that completed the study and those who were measured only at baseline (data not shown). 

The intervention program included the general exercise program used within the Swedish school physical education curriculum, supervised by the regular physical educational teachers but with the training increased to 40 minutes per day, corresponding to 200 minutes per week, but no specific registration was done as regard participation rate. Before the study the intervention school had had the same duration of physical education as the control schools. The duration was chosen in order to maximize a range of health-related benefits beyond just the gain in bone mass, which has been shown to respond to shorter bouts of body mass-bearing exercise [28–31]. Also, the physical education classes did not consist of any programs specifically designed to enhance muscle and bone mass. Instead, the classes included ordinary school physical education, both indoor and outdoor general physical activities such as a variety of ball games (e.g. basketball, handball, and soccer), running, jumping, and climbing activities (e.g. tag, rope climbing, and gymnastics-related activities on various apparatus). These activities were increased in duration so as not to bore the children with repeated standardized activities and to minimize the drop-out frequency frequently reported to occur with other study designs [32] and with the aim that any ordinary teacher in any school in the future could initiate such a program. The intensity level of the intervention program was moderate although it varied from low to high depending on if the current activity was more play-like or more of a competing situation. The aim of the intervention in a longer perspective was to increase general physical activity and not just exercise or training. In the control schools, the same type of activities were used, but as a compulsory Swedish school curriculum consisting of a mean 60 minutes per week given in one or two sessions per week.

A questionnaire, previously used in several pediatric studies but slightly modified for the POP study [21, 22, 33], was answered by the children together with the parents at baseline and follow-up. The questionnaire evaluated lifestyle factors such as socioeconomic and ethnic background, diseases, medications, fractures, consumption of dairy products, exclusion of anything in the diet, and physical activity in school and during leisure time (organized physical activity outside of school). Organized exercise outside of school was calculated as the weekly time (hours) spent in organized exercise (hours/week).

Body mass was measured with an Avery Berkel HL120 electric scale and stature by a wall-tapered Holtain Stadiometer with the children dressed in light clothes without shoes. Body mass index (BMI) was calculated as body mass/stature^2^ (kg/m^2^). A research nurse assessed the Tanner [34] staging. All were classified in Tanner stage 1 both at baseline and at follow-up.

Total body, arms, and legs lean tissue mass (kg) and fat mass (kg) were measured by dual-energy X-ray absorptiometry (DXA, DPX-L version 1.3 z, Lunar, Madison, WI) in a total body scan. All scans were performed by two research technicians who also analyzed the scans. The precision, evaluated by duplicate measurements in 13 healthy children aged 7–15 years (mean age 10 years), was 3.7% for total body fat mass and 1.5% for total body lean tissue mass.

Isokinetic peak torque of the right knee extensors and flexors were evaluated by a computerized dynamometer (Biodex System 3). Two physiotherapists performed the measurements. During the testing, the participants were seated with their hips flexed to 85° from the anatomical position. The axis of the knee was aligned with the Biodex axis of rotation. The participants were secured in the chair according to the standard Biodex procedure using shin, thigh, pelvic, and upper crossing torso stabilization straps. When required, a 10 cm thick pad was used to fill the space between the participant's back and the support of the chair. When the lever arm of the Biodex was longer than the lower leg of the participant a small pad was used to adjust for the difference. All participants were instructed to place their arms across their chest during the testing. The knee was positioned at 90° of flexion and went through a 75° range of motion, stopping at 15° of flexion. Concentric isokinetic knee extension and flexion peak torque were tested at an angular velocity of 60 and 180°/sec. Three submaximal trials were given prior to each testing velocities to assist with familiarization to the testing. In the literature, a familiarization to procedure prior to test is recommended although its validity has not been confirmed [35]. A total of five maximal repetitions (flexion and extension) at 60° sec were performed. After 30–60 seconds rest, 10 maximal repetitions at 180°/sec for both flexion and extension were done, with the highest peak torque (Nm) recorded for all measurements. All subjects received both visual and verbal encouragement during testing to ensure maximum effort at each velocity and repetition. Simple instructions were given to help the subjects understand the task. These instructions were given to each child when placed on the machine and included: “I want you to push and pull as hard and fast as you can 5/10 times. I will cheer you on and you can watch the screen to see how hard you are pushing.” Peak torque (Nm) at both 60° and 180°/sec for extension (PT_Ex60_; PT_Ex180_) and flexion (PT_Fl60_; PT_Fl180_) were normalized to body mass (kg) and expressed as Nm/kg. The torque data was corrected for gravity. A hard cushion setting of 1 was used and the data was not windowed. The intraindividual test variability, evaluated as the coefficient of variation for repeated measurements in 21 children, was 6.6% for PT_Ex60_, 12.1% for PT_Fl60_, 12.3% for PT_Ex180_, and 9.1% for PT_Fl180_. 

Vertical jump height (VJH), an estimation of neuromuscular performance, was used to assess physical performance. The vertical jump test was performed on an electronic mat connected to a digital timer that registered the total time in the air (Product name “Time It”; Eleiko Sport, Halmstad, Sweden). From this data, the height of the jump in centimeters was automatically calculated from the computer included in the standard equipment. All vertical jumps were performed from a standing position, and participants were first required to jump onto the mat with both feet and then make a maximal vertical jump. Each subject performed three vertical jumps from which the highest jump (cm) was recorded. The intraindividual test variability, evaluated as the coefficient of variation for repeated measurements in 21 children, was 5.9%.

Informed written consent was obtained from parents or guardians prior to participation. The study was approved by the Ethics Committee of Lund University. Data are presented as mean with standard deviation (SD) or 95% confidence interval (95% CI). All prospective data were converted into annual changes according to the following formula: [(follow-up data − baseline data)/the duration of follow-up] and expressed as the absolute or percentage change from baseline. Analyses of covariance (ANCOVA) were then used to compare the trait-specific annual changes in the groups, and baseline age and baseline peak torque values were included as covariates if there was a significant difference between the groups at baseline. Life style factors prior to and after study start were analysed with Fisher's exact test and Student *t*-test, respectively. Total mean duration of exercise during the study was defined as (school physical education and organized exercise outside of school) at baseline and at follow-up divided by two. Pearson's correlation coefficient was used to examine the relationship between the annual changes in muscle strength with the total mean duration of exercise.

The study design would detect a minimal difference of 0.123 Nm/kg in the annual change in muscle strength (PT_Ex60_) with 80% power and an alpha level of 0.05. The reason we chose PT_Ex60_ as our primary muscle force outcome is that there is evidence in the literature for a greater absolute increase in knee extensor compared to flexor muscle strength from age 9 to 21 years [36]. Training in children has also been reported to confer greater increase in knee extensor than in flexor peak torque [37].

## 3. Results

There were no differences at baseline in anthropometrics or body composition between the intervention and control group, with BMI reaching a borderline significant higher value in the intervention group (*P* = .05) (Table 1). Furthermore, there were no significant group differences at baseline in registered lifestyle factors. Before the intervention was initiated, there were no differences in total duration of physical activity, but when the intervention was initiated, the total duration of physical activity became greater in the intervention group than in the control group. At baseline, PT_Fl60_ and PT_Fl180_ (body mass-adjusted both *P* < .001) were both higher in the control group (Table 1). 

**Figure 1:**
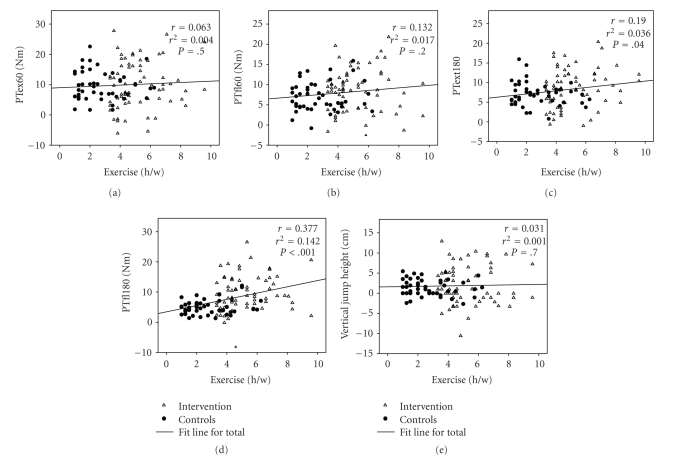
Correlation between total mean duration of exercise during the study and annual absolute gain in muscle strength (PT) and vertical jump height for interventions and controls.

**Figure 2:**
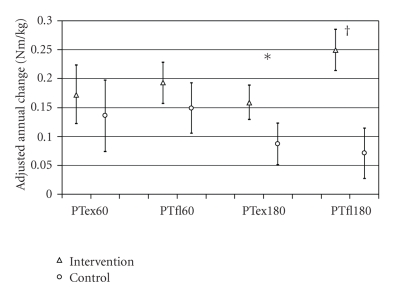
Mean, with 95% confidence interval, absolute annual changes in the body mass adjusted knee extension (ex), and flexion (fl) peak torque at 60 and 180°/sec in the intervention and control group. All data represent the annual changes adjusted for baseline age and the baseline values for that specific measurement, if there was a significant group difference at baseline. *P < .01, ^†^ *P* < .001 versus control group.

The annual gain in stature and body mass during the 12-month follow-up period was similar in the two groups whereas there was a greater increase in regional lean tissue mass and total body and regional fat mass in the intervention group (Table 1). After adjustment for baseline age and baseline peak torque values, if there was a significant difference at baseline between the groups, the annual changes in knee extension and flexion peak torque (PT_Ex180_ and PT_Fl180_) were significantly greater in the intervention than in the control group (Table 1). When peak torque was normalized to body mass, the gains in extension and flexor PT at 180 deg/sec were significantly greater in the intervention group compared with the control group (*P* < .01, adjusted for age at baseline and *P* < .001, adjusted for age and muscle strength at baseline, resp.) (Figure 2). Similar results were found when muscle strength was expressed relative to lean mass (data not shown). The total mean duration of exercise during the study period correlated with the annual gains in both PT_Ex180_ (*r* = 0.19, *P* < .05) and PT_Fl180_ (*r* = 0.38, *P* < .001) (Figure 1).

## 4. Discussion

This 12-month prospective, controlled, school-based exercise intervention study indicates that an increase in the duration of general moderately intense physical education in the school from 60 to 200 minutes per week is associated with an increased lower limb peak muscle strength gain in Prepubertal boys. A statistically significant difference between the groups can not automatically be transferred to a difference of biological and clinical significance; however, these findings still may have important public health implications as they provide evidence-based data to support the benefit of school-based physical education as an effective strategy to enhance muscular health in Prepubertal boys. 

There are several reasons why it is beneficial to enhance musculoskeletal health during growth. Bone density, muscle mass, and muscle strength are all traits that play an important role in reducing the risk of a number of chronic musculoskeletal diseases in adulthood [38, 39] and these traits are positively affected by physical activity [11–14, 21, 22, 28–31, 40]. In this study, we report that it is enough to increase moderate intense exercise when trying to improve muscle strength in young boys. These findings are consistent with the findings in girls in similar ages [20]. They also support other reports which conclude that 10 months of school-based exercise intervention in girls aged 9-10 years for 30 minutes 3 times per week is associated with 7 to 33% greater shoulder and knee extension isokinetic peak torque and grip strength, when the intervention group is compared with the control group [14]. To our knowledge no other studies have reported the effect of school-based intervention programs on muscle strength in prepubertal boys. However, extracurricular sports participation of 3 hours/week is positively associated with leg muscle force measured on a force plate [17], and with a handheld dynamometer [41]. Others report no correlation between physical activity and leg muscle strength in prepubertal boys [42, 43].

Several effects may explain the benefits seen in muscle strength from increased physical activity. Training may confer neuromuscular adaptations in conjunction with increases in muscle mass and muscle size, which all increase during puberty in association with the increased secretion of sex steroids [37, 44]. But the gain in muscle strength in response to training in Prepubertal children may also primarily be the result of neural adaptations as muscle strength could significantly increase without a concomitant augmentation in muscle size, even after a period and intensity of training for which muscle hypertrophy is evident in adults [8, 9, 44]. In our study, the increases in muscle strength were independent of the changes in lean tissue mass, suggesting that other factors than the mass of the muscle contribute to the increase in the force-producing capacity of muscles. It has been suggested that gain in muscle strength in response to resistance training during growth is the result of changes in neural factors, including enhanced motor unit activation, coordination, recruitment, and/or firing frequency [7, 8, 44, 45]. However, whether the increase in muscle strength in children after a moderate intensity exercise program also can be accounted for by these neural adaptations is still unclear. Furthermore, changes in limb length and subsequently the muscle moment arm are also factors contributing to improvements in torque production as isokinetic peak torque and limb length are closely related [36, 46].

Published studies that examined the effect of resistance training on muscle hypertrophy in children are usually short studies over weeks or months and studies that usually rely on limb circumference when estimating muscle mass or muscle size [7–9, 37]. The DXA technique, however, allows us to measure the mass of the lean tissue, which has shown to be comparable to *in vivo* techniques of measuring muscle mass [47, 48]. We observed that the 12-month school-based physical activity program was associated with a trend of gaining more lean mass in all regions, being significant only in the arms, relative to the control group (Table 1). These findings are consistent with the results of a 6-year prospective study reporting that boys in the upper quintile of habitual physical activity gain 3–6% more lean mass than those in the lowest quintile [11, 49]. Given that the boys in our study did not participate in a specific resistance training program, it seems that the exercise-induced gains in lean tissue mass were related to the relatively large increase in the duration of training (from 60 to 200 minutes per week) coupled with the follow-up period of 12 months. The finding of a correlation between total duration of physical activity and changes in muscle strength further supports the view that there really is a causal relationship between the duration of habitual physical activity and the gain in muscle strength in Prepubertal boys, even if it must be emphasised that the determination coefficient (*r* ^2^) indicates that the duration of physical activity during the study explained no more than 3.6%–14.4% of the variance in the reported traits (Figure 1). 

Despite reports that general physical training can improve muscle strength in children, there are conflicting reports as to whether these benefits translate into improvements in other physical performance estimates such as athletic performance, VJH, long jump, or sprint speed [7, 9, 50, 51]. When we examined the exercise induced effects on VJH in this study, the gain in the exercise group was in absolute values an improvement relative to the control group although the difference was not statistically significant. This lack of statistical significance is consistent with previous reports, inferring that school-based exercise intervention is associated with a nonsignificant difference but a trend toward a greater improvement in the exercise compared to control girls for both VJH (~10%, *P* = .14) and long jump (~3%, *P* = .10) [29]. The lack of a significant difference in VJH in our study could be explained by the nonspecific nature of the training program and/or the inability of the test to accurately capture changes in functional performance. Since changes in VJH are believed to reflect neuromuscular adaptations [52], it could be that the activities provided in our intervention did not specifically replicate the vertical jump movement pattern and thus were not sufficient to significantly improve jumping performance. 

An unexpected finding in our study was the greater annual gain in arm fat mass in the intervention group compared with the controls (Table 1). However, others have also reported an increase in fat mass in children associated with increased physical training [20, 53]. The greater gain in fat mass in the intervention group in the present study could be explained by an increased food intake accompanying the increased training. Although we did not assess dietary habits, we have previously reported that the discrepancy in fat gain was most likely the result of other influences besides physical activity, because there was no dose-response relationship between the duration of exercise and gain in fat mass [21].

There are limitations to this study. This was not a randomized, controlled study, as randomization was refused by the principals, teachers, parents, and children since it was neither feasible nor practical for some children to be given additional exercise during compulsory school hours while others were not. But since all schools had a similar amount of regular school physical education before study start and since there were no differences in anthropometry between participants and nonparticipants or between participants and dropouts, the risk of selection bias seems minimal. Due to lack of resources in our research laboratory, the control group boys were not remeasured until after two years. However, as all the boys remained Prepubertal during the study, it was possible to compare the annual changes between the groups as the development of muscle strength is proportional to the gains in stature and body mass that occur linearly during this period [54, 55]. Third, the estimate of lean tissue mass and fat mass was made using DXA. Even though this technique is comparable to criterion *in vivo* techniques [47, 48], it has been shown that DXA-based body composition measures during growth can be influenced by differences in hydration status, body size, and fat distribution [56]. Another weakness is that we do not have measurements of leg length as changes in limb length could influence the muscle moment arm in relation to the improvement in torque production. Also, during the different breaks from school, there was no intervention given, a fact that if anything would reduce our estimated effect of the intervention program, due to possible detraining in the intervention group [50]. Finally, physical activity habits were assessed by questionnaire and limited to organized exercise only. 

In conclusion, increasing the amount of moderately intense exercise within the school curriculum physical education to 40 minutes per day provides a feasible strategy to enhance muscle strength in Prepubertal boys. These findings have important clinical implications, as the first two decades in life may represent the most opportune time to reduce the risk of a number of chronic musculoskeletal health conditions [38]. Thus, the findings of this study support the notion that health benefits through increased school-based exercise for young boys can be achieved without adding external resources, costs, personnel, financial, or spatial resources to the school budget.

## Figures and Tables

**Table 1:** Baseline data, annual unadjusted absolute changes, and adjusted difference in anthropometry, body composition, muscle strength, and vertical jump height in the intervention and control group.

	Baseline			Annual Changes			
	Intervention (*n* = 68)	Controls (*n* = 46)	*P*-value	Intervention (*n* = 68)	Controls (*n* = 46)	Adjusted difference (95% CI)	*P*-value
Age (years)	7.8 ± 0.5	7.9 ± 0.6	.23	—	—	—	
*Anthropometry*							
Stature (cm)	129.4 ± 6.3	129.5 ± 6.7	.48	5.6 (5.4, 5.9)	5.7 (5.5, 5.9)	−0.07 (−0.41, 0.27)	.70
Body mass (kg)	27.8 ± 4.7	26.8 ± 4.6	.08	3.0 (2.7, 3.3)	3.1 (2.8, 3.4)	−0.06 (−0.49, 0.37)	.79
BMI (kg/m^2^)	16.5 ± 1.8	15.9 ± 1.7	.05	0.3 (0.2, 0.5)	0.3 (0.2, 0.5)	−0.03 (−0.24, 0.19)	.81
Total body fat (%)	13.2 ± 6.8	12.6 ± 5.4	.52	2.1 (1.5, 2.7)	1.3 (0.8, 1.7)	0.96 (0.15, 1.76)	**.02**
*Lean body mass (kg)*							
Total body	21.70 ± 2.74	21.50 ± 2.90	.24	2.28 (2.13, 2.43)	2.13 (2.00, 2.25)	0.15 (−0.06, 0.35)	.17
Legs	6.78 ± 1.15	6.68 ± 1.23	.16	1.00 (0.93, 1.06)	0.96 (0.90, 1.02)	0.03 (−0.06, 0.13)	.46
Arms	1.80 ± 0.35	1.81 ± 0.38	.65	0.30 (0.26, 0.34)	0.19 (0.17, 0.22)	0.11 (0.06, 0.16)	<**.001**
*Fat mass (kg)*							
Total body	3.56 ± 2.59	3.31 ± 2.01	.44	1.14 (0.88, 1.39)	0.81 (0.59, 1.03)	0.37 (0.01, 0.72)	**.04**
Legs	1.61 ± 0.96	1.46 ± 0.75	.26	0.43 (0.33, 0.53)	0.36 (0.27, 0.45)	0.08 (−0.06, 0.23)	.26
Arms	0.30 ± 0.31	0.34 ± 0.31	.61	0.14 (0.09, 0.18)	0.02 (−0.02, 0.04)	0.12 (0.07, 0.18)	<**.001**
*Peak Torque—unadjusted (Nm)*							
PT_Ex60_	43.0 ± 9.9	43.4 ± 10.7	.56	10.2 (8.3, 12.1)	9.8 (8.3, 11.2)	0.74 (−1.79, 3.27)	.56
PT_Fl60_	21.4 ± 6.4	24.4 ± 6.7	**.04**	8.6 (7.4, 9.9)	7.0 (6.0, 8.1)	1.66 (−0.15, 3.48)	.07
PT_Ex180_	35.1 ± 7.7	35.4 ± 7.7	.47	8.7 (7.5, 9.9)	7.0 (6.2, 7.9)	1.77 (0.20, 3.35)	**.03**
PT_Fl180_	19.0 ± 4.9	22.6 ± 5.2	<**.001**	9.7 (8.3, 11.1)	5.0 (4.3, 5.8)	4.87 (2.94, 6.80)	<**.001**
*Peak Torque—body mass-adjusted (Nm/kg)*							
PT_Ex60_	1.54 ± 0.22	1.61 ± 0.27	.26	0.17 (0.11, 0.23)	0.14 (0.10, 0.18)	0.04 (−0.04, 0.12)	.37
PT_Fl60_	0.77 ± 0.18	0.91 ± 0.20	**<.001**	0.21 (0.17, 0.25)	0.13 (0.09, 0.16)	0.04 (−0.01, 0.10)	.14
PT_Ex180_	1.26 ± 0.17	1.32 ± 0.19	.25	0.16 (0.12, 0.19)	0.09 (0.07, 0.11)	0.07 (0.02, 0.12)	**.003**
PT_Fl180_	0.69 ± 0.15	0.84 ± 0.11	**<.001**	0.25 (0.20, 0.29)	0.07 (0.05, 0.10)	0.14 (0.08, 0.21)	**<.001**
*Physical performance *							
Vertical jump height (cm)	24.2 ± 4.7	24.8 ± 4.3	.90	2.1 (1.0, 3.2)	1.3 (0.7, 1.9)	0.56 (−0.86, 1.97)	.44

The baseline values are presented as unadjusted means ± SD and the annual changes as unadjusted means with 95% confidence interval (95% CI). All baseline comparisons are adjusted for age at baseline. The differences between the annual changes in the intervention and the control group are presented adjusted for differences in age at baseline and, if there was a significant group difference at baseline, the baseline values for that specific measurement (accounts for PT_EX60_, PT_EX180_, PT_Fl60_ and PT_Fl180_).
